# Simultaneous deactivation of FAK and Src improves the pathology of hypertrophic scar

**DOI:** 10.1038/srep26023

**Published:** 2016-05-16

**Authors:** Linlin Su, Xiaodong Li, Xue Wu, Bo Hui, Shichao Han, Jianxin Gao, Yan Li, Jihong Shi, Huayu Zhu, Bin Zhao, Dahai Hu

**Affiliations:** 1 Department of Burns and Cutaneous Surgery, Xijing Hospital, the Fourth Military Medical University, Xi’an, Shaanxi 710032, China; 2 Department of Burns and Plastic Surgery, General Hospital of Lanzhou Petrochemical Company, Lanzhou, Gansu 730060, China; 3 Department of Surgery, the Second Affiliated Hospital, School of Medicine, Xi’an Jiaotong University, Xi’an, Shaanxi 710004, China

## Abstract

Hypertrophic scar (HS) is a serious fibrotic skin condition with currently no satisfactory therapy due to undefined molecular mechanism. FAK and Src are two important non-receptor tyrosine kinases that have been indicated in HS pathogenesis. Here we found both FAK and Src were activated in HS *vs.* normal skin (NS), NS fibroblasts treated with TGF-β1 also exhibited FAK/Src activation. Co-immunoprecipitation and dual-labelled immunofluorescence revealed an enhanced FAK-Src association and co-localization in HS *vs.* NS. To examine effects of FAK/Src activation and their interplay on HS pathogenesis, site-directed mutagenesis followed by gene overexpression was conducted. Results showed only simultaneous overexpression of non-phosphorylatable mutant FAK Y407F and phosphomimetic mutant Src Y529E remarkably down-regulated the expression of Col I, Col III and α-SMA in cultured HS fibroblasts, alleviated extracellular matrix deposition and made collagen fibers more orderly in HS tissue *vs.* the effect from single transfection with wild-type or mutational FAK/Src. Glabridin, a chemical found to block FAK-Src complex formation in cancers, exhibited therapeutic effects on HS pathology probably through co-deactivation of FAK/Src which further resulted in FAK-Src de-association. This study suggests FAK-Src complex could serve as a potential molecular target, and FAK/Src double deactivation might be a novel strategy for HS therapy.

Hypertrophic scarring (HS) is a common fibro-proliferative disorder that typically follows burns and other injuries involving the deep dermis which is predominantly occupied by the overgrown dermal fibroblasts and the excessive extracellular matrix (ECM)^1^. The formation and development of HS are closely accompanied with the extravagant secretion of various cytokines, such as TGF-β^2^,^3^, which is suggested to stimulate the growth of granulation tissues and promote the transformation of fibroblasts into proliferative and more contractile myofibroblast-type cells^4^, which is identified by the expression of α-smooth muscle actin (α-SMA) and in turn secretes abundant ECM proteins, including fibrous collagens that cause scar formation and organ fibrosis. However, the existing therapies for HS are rather restricted largely because of the quite limited understanding of the underlying mechanism and involved signaling pathway.

Focal adhesion kinase (FAK) and Src are two important non-receptor tyrosine kinases that have been indicated to be involved in the process of would healing^5^,^6^,^7^. FAK is found at focal adhesions, the sites of integrin clustering at the cell–ECM interface, where it provides both signaling and scaffolding functions^8^. FAK is activated in a range of tumor cells, and its increased activity correlates with the malignancy and invasiveness of various tumors^9^,^10^. Recent studies have implied FAK as a central mediator of fibrogenesis, and highlighted this kinase as a potential therapeutic target in fibrotic diseases^11^, for instance, the inhibition of FAK might represent a novel therapy for scarring disorders^12^.

The cellular form of the *Src* gene (*c-Src*) is the first proto-oncogene that was first discovered in the vertebrate genome in 1970s^13^. The coding product of *c-Src* is Src and its activity is regulated by tyrosine phosphorylation at two different residues with opposing effects. Phosphorylation at Tyr^529^ deactivates Src, while phosphorylation at Tyr^416^ results in Src activation^14^. Recent studies have showed that Src was activated in cells along the wounding edge of cultured mouse corneal epithelial cells^15^, and it integrated early wound responses and late epimorphic regeneration in zebrafish^16^, suggesting Src might play a significant role in various stages of wound healing. However, its involvement in HS pathogenesis has less been investigated.

FAK and Src often work as a functional protein complex in various tissues and cells. FAK-Src complex-mediated signaling pathways are normally required in tumor progression. For instance, inhibiting the formation of FAK-Src complex by glabridin was found to reduce cancer cell proliferation and motility^17^. To some extent, HS is considered as a kind of benign skin tumor due to its excessive and rapid cell overgrowth, however, if FAK-Src protein complex exists and exerts roles in HS remains to be clarified. In addition, if the regulation of the activated status of FAK-Src complex would affect HS formation, development and progress also needs to be investigated.

In this study, we first evaluated the active status of FAK and Src in HS *vs*. normal skin (NS) both *in vivo* and *in vitro* by assessing relative expression levels of activating forms *p*-FAK-Tyr^407^ and *p*-Src-Tyr^416^, as well as deactivating form *p*-Src-Tyr^529^. Then we compared the difference on FAK-Src interaction between HS and NS by co-immunoprecipitation and dual-labelled immunofluorescence. Next we performed site-directed mutagenesis followed by gene overexpression to suppress the activity of FAK or Src in HS and tried to examine if regulating FAK/Src activity would improve HS pathology. Finally, glabridin that has been reported to inhibit FAK-Src complex formation in cancer cells was utilized to further confirm the importance of FAK/Src activation on HS pathogenesis.

## Results

### Both FAK and Src are activated in hypertrophic scar-derived dermis *in vivo*

**Figure 1:**
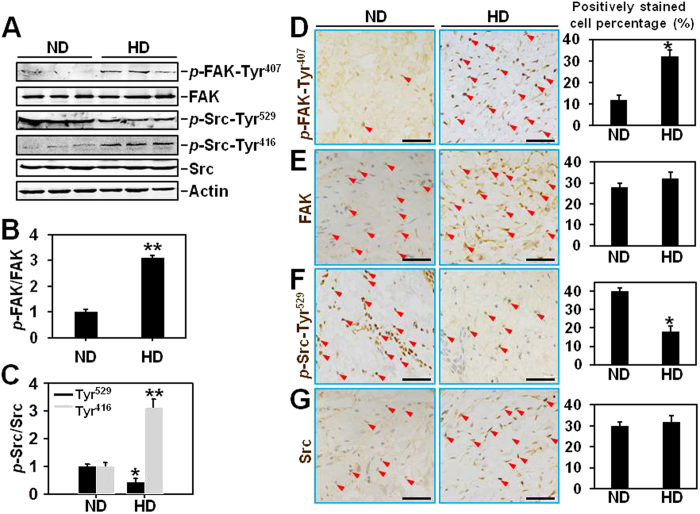
The protein expression and tissue distribution of *p*-FAK-Tyr^407^, FAK, *p*-Src-Tyr^529^, *p*-Src-Tyr^416^ and Src in normal skin and hypertrophic scar-derived dermis. (**A**) Immunoblots show the expression of *p*-FAK-Tyr^407^, FAK, *p*-Src-Tyr^529^, *p*-Src-Tyr^416^ and Src in lysates from normal skin dermis (ND) and hypertrophic scar dermis (HD). Actin serves as an equal loading control. (**B,C**) Bar graphs compare the difference on relative protein levels of *p*-FAK-Tyr^407^ (**B**), *p*-Src-Tyr^529^ and *p*-Src-Tyr^416^ (**C**) between ND and HD. Each data point of the phosphorylated form was normalized against its corresponding total protein level with the value in ND group arbitrarily set at 1. Each bar represents a mean ± SD of *n* = 4. ^*^P < 0.05, ^**^P < 0.01. (**D–G**) Immunohistochemistry was performed on paraffin-embedded normal skin and hypertrophic scar sections to locate *p*-FAK-Tyr^407^ (**D**), FAK (**E**), *p*-Src-Tyr^529^ (**F**) and Src (**G**) in the dermal portion. Immunoreactive *p*-FAK-Tyr^407^, FAK, *p*-Src-Tyr^529^ and Src are shown as *brownish* precipitates as indicated by *red* arrowheads. Scale bars = 50 μm. Bar graphs on the right column compare the difference on *p*-FAK-Tyr^407^- (**D**), FAK- (**E**), *p*-Src-Tyr^529^- (**F**) and Src- (**G**) positively stained cell percentage between ND and HD. 200 fibroblasts per tissue section (*n* = 4) are counted in each group. ^*^P < 0.05.

The protein levels of FAK, Src, and their corresponding phosphorylated forms *p*-FAK-Tyr^407^, *p*-Src-Tyr^529^ and *p*-Src-Tyr^416^ between normal skin dermis (ND) and hypertrophic scar dermis (HD) were first compared (Fig. 1A–C). Results showed that the total protein levels of FAK and Src were the same between ND and HD (Fig. 1A), the relative levels of activating froms *p*-FAK-Tyr^407^ and *p*-Src-Tyr^416^ were significantly up-regulated by ~3-fold (Fig. 1B,C), while the deactivating form *p*-Src-Tyr^529^ was down-regulated by ~50% (Fig. 1C) in HD, indicating the activation of both FAK and Src in HD. Immunohistochemistry was then performed to evaluate tissue distribution/localization of *p*-FAK-Tyr^407^ (Fig. 1D), FAK (Fig. 1E), *p*-Src-Tyr^529^ (Fig. 1F) and Src (Fig. 1G) in ND and HD. Results showed that both FAK- (Fig. 1E) and Src- (Fig. 1G) positively stained fibroblast percentages remained the same (~30%) between ND and HD, while *p*-FAK-Tyr^407^- (Fig. 1D) and *p*-Src-Tyr^529^- (Fig. 1G) positively stained fibroblast rates in HD were remarkably up-regulated to ~30% *vs.* ~10% in ND or down-regulated to ~20% *vs.* ~40% in ND, respectively. These IHC results were consistent with immunoblotting data.

### Both FAK and Src are activated in hypertrophic scar-derived fibroblasts *in vitro*

**Figure 2:**
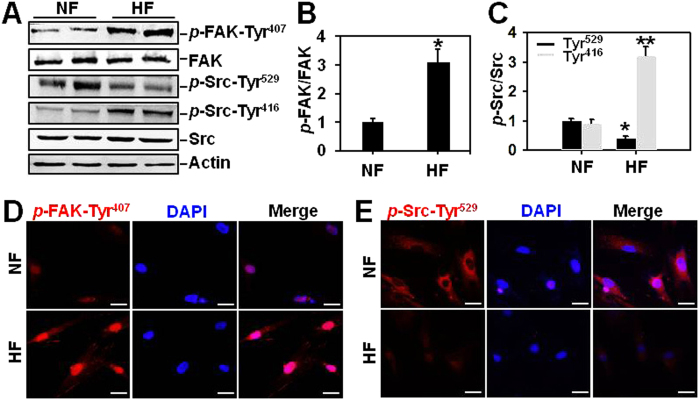
The protein expression and cellular distribution of *p*-FAK-Tyr^407^, FAK, *p*-Src-Tyr^529^, *p*-Src-Tyr^416^ and Src in normal skin and hypertrophic scar-derived fibroblasts. (**A**) Immunoblots show the expression of *p*-FAK-Tyr^407^, FAK, *p*-Src-Tyr^529^, *p*-Src-Tyr^416^ and Src in lysates from normal skin fibroblasts (NF) and hypertrophic scar fibroblasts (HF). Actin serves as an equal loading control. (**B,C**) Bar graphs compare the difference on relative protein levels of *p*-FAK-Tyr^407^ (**B**), *p*-Src-Tyr^529^ and *p*-Src-Tyr^416^ (**C**) between NF and HF. Each data point of the phosphorylated form was normalized against its corresponding total protein level with the value in NF group arbitrarily set at 1. Each bar represents a mean ± SD of *n* = 4. ^*^P < 0.05, ^**^P < 0.01. (**D,E**) Immunocytofluorescence analysis was performed in cultured NF and HF to visualize the fluorescence intensity of *p*-FAK-Tyr^407^ (**D**) and *p*-Src-Tyr^529^ (**E**), as well as their cellular localization. Immunoreactive proteins were shown in *red* fluorescence. Nuclei were stained with DAPI (*blue*). Scale bars = 25 μm.

The protein expression of FAK, Src, and their corresponding phosphorylated forms *p*-FAK-Tyr^407^, *p*-Src-Tyr^529^ and *p*-Src-Tyr^416^ in normal skin fibroblasts (NF) and hypertrophic scar fibroblasts (HF) were examined (Fig. 2A–C). Results showed that total protein levels of FAK and Src were the same between NF and HF (Fig. 2A), the relative levels of activating froms *p*-FAK-Tyr^407^ and *p*-Src-Tyr^416^ were significantly up-regulated by ~3-fold (Fig. 2B,C), while the deactivating form *p*-Src-Tyr^529^ was down-regulated by ~60% (Fig. 2C) in HF, indicating the activation of both FAK and Src in HF, which coincides with *in vivo* data in Fig. 1. Immunocytofluorescence was used to further reflect the fluorescence intensity and cellular localization of *p*-FAK-Tyr^407^ (Fig. 2D) and *p*-Src-Tyr^529^ (Fig. 2E) in NF and HF. Results showed that *p*-FAK-Tyr^407^ immunostaining was predominantly detected at nuclei in both NF and HF with HF displaying much more intensive staining than NF (Fig. 2D). In contrast, *p*-Src-Tyr^529^ exhibited cytoplasm staining in both NF and HF with much stronger staining in NF than that in HF (Fig. 2E).

### Both FAK and Src are activated upon TGF-β1 stimulation in cultured normal skin-derived fibroblasts

**Figure 3:**
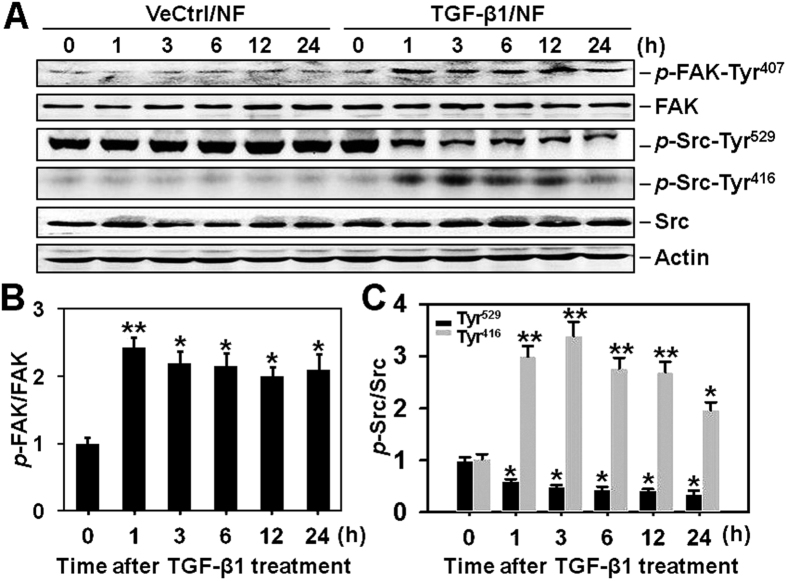
The protein expression of *p*-FAK-Tyr^407^, FAK, *p*-Src-Tyr^529^, *p*-Src-Tyr^416^ and Src following TGF-β1 treatment in normal skin fibroblasts. (**A**) Immunoblots show the expression of *p*-FAK-Tyr^407^, FAK, *p*-Src-Tyr^529^, *p*-Src-Tyr^416^, and Src in normal skin fibroblasts (NF) following TGF-β1 (10 ng/ml) or its vehicle control (VeCtrl, 10 mM citric acid) treatment at designated time points of 0, 1, 3, 6, 12 and 24 h. Actin serves as an equal loading control. (**B,C**) Bar graphs show changes on relative protein levels of *p*-FAK-Tyr^407^ (**B**), *p*-Src-Tyr^416^ and *p*-Src-Tyr^529^ (**C**) after TGF-β1 treatment over time. Each data point of the phosphorylated protein was normalized against its corresponding total protein level with the value at 0 h in TGF-β1 group arbitrarily set at 1. Each bar represents a mean ± SD of *n* = 4. ^*^P < 0.05, ^**^P < 0.01.

TGF-β1 is known as a key regulatory cytokine involved in hypertrophic scar pathogenesis, here we tried to investigate whether TGF-β1 stimulation would affect the expression of *p*-FAK-Tyr^407^, FAK, *p*-Src-Tyr^529^, *p*-Src-Tyr^416^ and Src in normal skin fibroblasts. Immunoblotting results showed that both FAK and Src levels did not change after TGF-β1 (5 ng/ml) treatment (Fig. 3A), while the relative expression of *p*-FAK-Tyr^407^ (Fig. 3B) and *p*-Src-Tyr^416^ (Fig. 3C), as well as *p*-Src-Tyr^529^ (Fig. 3C) were significantly up- or down-regulated, respectively. These changes started as early as 1 h post-TGF-β1 treatment and lasted up to 24 h post-treatment (Fig. 3A–C). These *in vitro* findings further verified results in Figs 1 and 2.

### The functional protein complex FAK-Src is activated in hypertrophic scar

**Figure 4:**
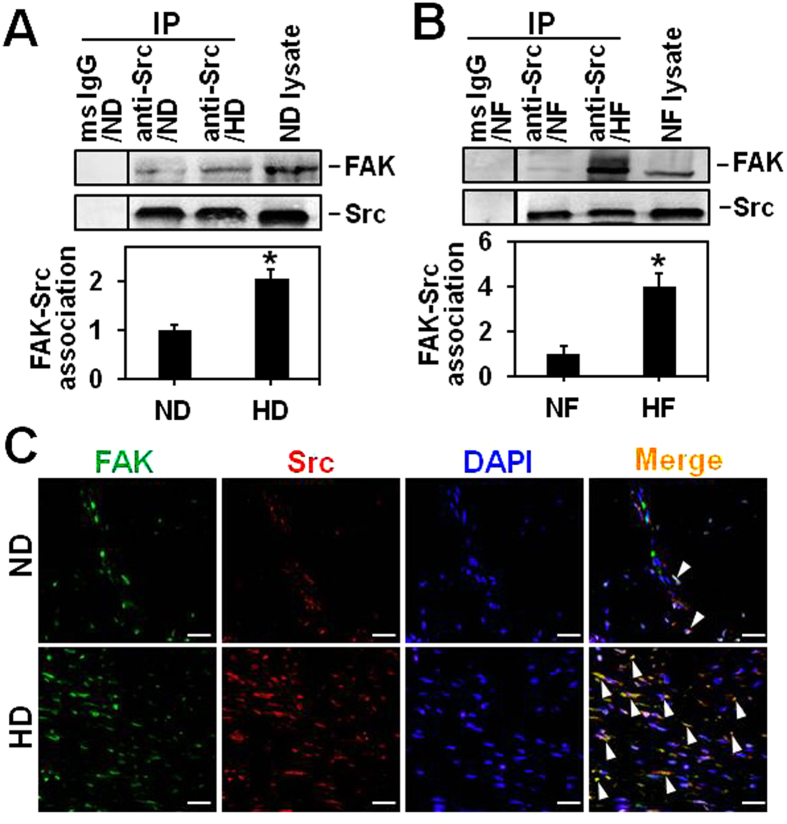
The association of FAK with Src in normal skin and hypertrophic scar *in vivo* and *in vitro*. (**A,B**) Co-immunoprecipitation was performed to compare the difference on FAK-Src association between ND and HD (**A**), as well as between NF and HF (**B**). The presence of FAK with Src in the immunoprecipitated protein complexes was then detected by immunoblotting. Mouse origin anti-Src IgG was used as the precipitating antibody to extract the interacting proteins binding to Src. Normal mouse (ms) IgG was used to replace anti-Src IgG and served as negative control, while lysates from ND (**A**) or NF (**B**) without co-immunoprecipitation served as positive control. Bar graphs compare the difference on FAK-Src association between ND and HD (***A***), as well as between NF and HF (**B**). Each data point of FAK was normalized against its corresponding Src level with the value in ND (**A**) or NF (**B**) arbitrarily set at 1. Each bar represents a mean ± SD of *n* = 4. ^*^P < 0.05, ^**^P < 0.01. (**C**) Dual-labelled immunohistofluorescence analysis was performed on paraffin-embedded normal skin and hypertrophic scar sections to co-locate FAK (*green*) with Src (*red*) in the dermal portion. Corresponding images were merged, *white* arrowheads pointed to areas of co-localization (*orange*). Nuclei were stained with DAPI (*blue*). Scale bars = 40 μm.

FAK and Src were known to form a functional protein complex in various cells and tissues^18^. In this study, we found FAK was structurally associated with Src in both ND (Fig. 4A) and NF (Fig. 4B), while their interaction was remarkably induced by ~2- or ~4-fold in HD (Fig. 4A) and HF (Fig. 4B), respectively. Dual-labelled immunofluorescence analysis showed that the co-localization (as shown in *orange* in merged images) of FAK with Src was much stronger in HD (*lower* panel in Fig. 4C) than that in ND (*upper* panel in Fig. 4C). These results support the notion that FAK and Src could form FAK-Src protein complex under both normal and pathological skin conditions by protein–protein interaction. In addition, the differential FAK-Src interaction between normal skin and hypertrophic scar as our results shown may suggest that FAK-Src complex is crucial in the regulation of scar formation and progression.

### Simultaneous deactivation of FAK and Src by co-overexpressing FAK and Src mutants significantly down-regulates the expression of Col I, Col III and a-SMA in cultured HS fibroblasts

**Figure 5:**
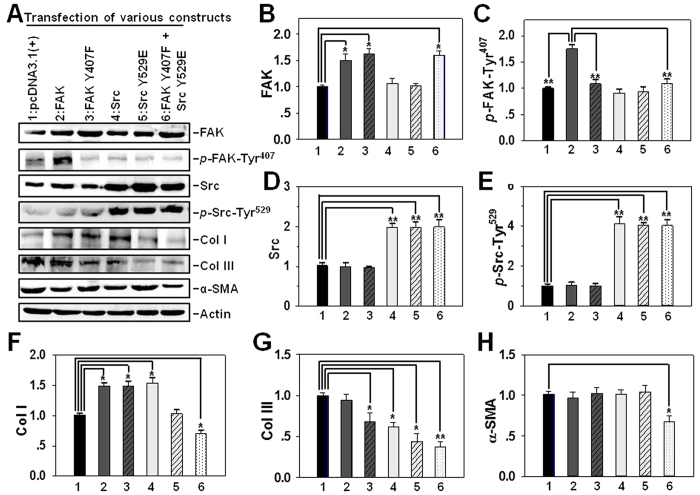
The effect of transfection of hypertrophic scar fibroblasts with various FAK and/or Src constructs on the steady-state protein levels of Col I, Col III and α-SMA. (**A**) Immunoblotting analysis on the expression of FAK, *p*-FAK-Tyr^407^, Src, *p*-Src-Tyr^529^, Col I, Col III and α-SMA following transfection of hypertrophic scar fibroblasts with eukaryotic expression vector pcDNA3.1(+) alone *vs*. various FAK or Src wide-type or mutational constructs. Fibroblasts cultured between 3^rd^ to 4^th^ sub-passages were transfected with plasmid DNA, cell lysates were harvested three days thereafter and subjected to immunoblotting. Actin served as an equal protein loading control. (**B–H**) Bar graphs summarize immunoblotting results of FAK (**B**), *p*-FAK-Tyr^407^ (**C**), Src (**D**), *p*-Src-Tyr^529^ (**E**), Col I (**F**), Col III (**G**) and□α-SMA□(**H**) from three independent experiments. Each data point was normalized against its corresponding actin level with the value in pcDNA3.1(+) alone group arbitrarily set as 1. Each bar represents a mean ± SD of *n* = 5. ^*^P < 0.05, ^**^P < 0.01 *vs*. pcDNA3.1(+) alone (**B,D–H**) or pcDNA3.1(+)/FAK group (**C**). Lane 1: pcDNA3.1(+), lane 2: pcDNA3.1(+)/FAK, lane 3: pcDNA3.1(+)/FAK Y407F, lane 4: pcDNA3.1(+)/Src, lane 5: pcDNA3.1(+)/Src Y529E, lane 6: pcDNA3.1(+)/FAK Y407F + pcDNA3.1(+)/Src Y529E.

Having demonstrated that the expression of *p*-FAK-Tyr^407^ and *p*-Src-Tyr^529^ was notably up-regulated and down-regulated in hypertrophic scar, respectively, site-directed mutagenesis followed by gene overexpression was conducted to examine the function of FAK at Tyr^407^ and Src at Tyr^529^ in scar pathogenesis. Non-phosphorylatable FAK mutant at Tyr^407^ (FAK Y407F) and phosphomimetic Src mutant at Tyr^529^ (Src Y529E) were constructed by site-directed mutagenesis in which the Tyr residue was replaced by Phe (Y407F) or Glu (Y529E), respectively. These mutants were then overexpressed in hypertrophic scar-derived fibroblasts between 3^rd^ to 4^th^ sub-passages, with the empty vector pcDNA3.1(+) or wild-type FAK or Src serving as control. Immunoblotting analysis showed an increase of 50% ~ 70% in FAK protein level when cells were transfected with plasmid containing wild-type or mutational FAK *vs.* empty vector (Fig. 5A,B, *lane 2, 3, 6 vs. lane 1*), while transfection with wild-type or mutational Src did not affect FAK level compared to empty vector (Fig. 5A,B, *lane 4, 5 vs. lane 1*). As expected, transfection with wild-type FAK significantly up-regulated *p*-FAK-Tyr^407^ level by ~75% *vs.* empty vector (Fig. 5A,C, *lane 2 vs. lane 1*), while transfection with plasmid containing FAK Y407F mutant resulted in remarkable decrease of *p*-FAK-Tyr^407^ level *vs*. wild-type FAK (Fig. 5A,C, *lane 3, 6 vs. lane 2*). Also, transfection with wild-type or mutational Src did not affect *p*-FAK-Tyr^407^ level compared to empty vector (Fig. 5A,C, *lane 4, 5 vs. lane 1*). On the other hand, overexpression of wild-type Src or Src Y529E mutant induced a 2-fold increase in Src level (Fig. 5A,D, *lane 4, 5, 6 vs. lane 1*) or a 4-fold increase in *p*-Src-Tyr^529^ level (Fig. 5A,E, *lane 4, 5, 6 vs. lane 1*) *vs.* empty vector, respectively, while transfection with wild-type or mutational FAK did not affect either Src (Fig. 5A,D, *lane 2, 3 vs. lane 1*) or *p*-Src-Tyr^529^ protein level (Fig. 5A,E, *lane 2, 3 vs. lane 1*) compared to empty vector. It was noted that overexpression of either wild-type or mutational Src could dramatically increase *p*-Src-Tyr^529^ level *vs*. wild-type Src (Fig. 5A,E*, line 5, 6 vs. lane 4*). Importantly, only simultaneous deactivation of FAK and Src by co-transfection of HS fibroblasts with FAK Y407F and Src Y529E was able to significantly decrease all three ECM protein levels: Col I (Fig. 5A,F*, lane 6 vs. lane 1*), Col III (Fig. 5A,G*, lane 6 vs. lane 1*) and α-SMA (Fig. 5A,H*, lane 6 vs. lane 1*) compared to empty vector, although single transfection of FAK Y407F, wild-type Src or Src Y529E also reduced Col III expression level to some extent compared to empty vector (Fig. 5A,G*, lane 3, 4, 5 vs. lane 1*). Unexpectedly, single transfection with FAK, FAK Y407F or wild-type Src even up-regulated Col I level compared to empty vector (Fig. 5A,F*, lane 2, 3, 4 vs. lane 1*).

### Simultaneous deactivation of FAK and Src improves extracellular matrix deposition and collagen fiber arrangement in hypertrophic scar tissue

**Figure 6:**
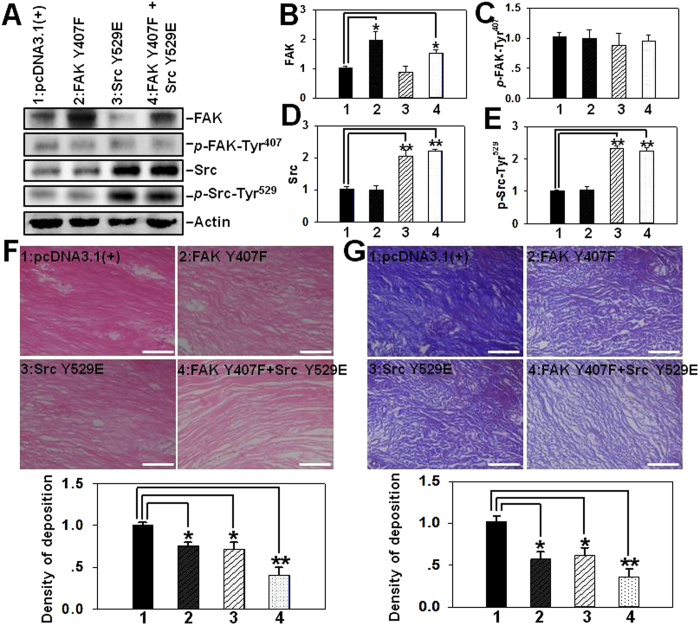
The effect of transfection of hypertrophic scar tissue with various FAK and/or Src mutants on extracellular matrix deposition and collagen fiber arrangement. Hypertrophic scar tissues were transfected with plasmid DNA of pcDNA3.1(+) vector alone *vs*. various FAK and/or Src mutants. Seven days thereafter scar samples were either subjected to immunoblotting (**A**), or paraffin-embedded and sectioned at 4-μm and processed for H&E (**F**) or Masson staining (**G**). (**A**) Immunoblotting analysis on the expression of FAK, *p*-FAK-Tyr^407^, Src and *p*-Src-Tyr^529^ following transfection of various constructs. (**B–E**) Bar graphs summarize immunoblotting results of FAK (**B**), *p*-FAK-Tyr^407^ (**C**), Src (**D**) and *p*-Src-Tyr^529^ (**E**) from three independent experiments. Each data point was normalized against its corresponding actin level with the value in pcDNA3.1(+) alone group arbitrarily set as 1. Each bar represents a mean ± SD of *n* = 5. ^*^P < 0.05, ^**^P < 0.01 *vs*. pcDNA3.1(+) alone. (**F**) In H&E staining, the cytoplasm and extracellular matrix, such as collagens, were stained with eosin and shown in *pink*, while the nuclei were counterstained with hematoxylin and shown in *blue*. Scale bar = 100 μm. The relative density of ECM deposition was analyzed by Image J software with the value in pcDNA3.1(+) group arbitrarily set as 1. Each bar represents a mean ± SD of *n* = 4. ^*^P < 0.05, ^**^P < 0.01 *vs*. pcDNA3.1(+) group. (**G**) In Masson staining, collagen fibers were shown in *blue*, the cytoplasm was shown in *pink*, whereas nuclei were shown in *dark blue*. The arrangement of collagen fiber in each treatment group was observed. Scale bar = 100 μm. The relative density of collagen deposition was analyzed by Image J software with the value in pcDNA3.1(+) group arbitrarily set as 1. Each bar represents a mean ± SD of *n* = 4. ^*^P < 0.05, ^**^P < 0.01 *vs.* pcDNA3.1(+) group.

Various FAK or Src constructs were transfected into HS tissue, and immunoblotting was first performed to confirm the successful transfection *in vivo*. Results showed that Transfection with plasmids containing FAK mutant up-regulated total FAK protein level and transfection with plasmids containing Src mutant up-regulated both Src and *p*-Src-Tyr^529^ protein levels compared to endogenous FAK or Src level (Fig. 6A–E), further demonstrating the effective transfection *in vivo*. H&E staining was then conducted to observe the effect of single or simultaneous transfection with FAK and/or Src mutants on extracellular matrix deposition. Results revealed that scar tissue transfected with empty vector showed excessive ECM deposition (Fig. 6F), which is consistent with the pathological feature of hypertrophic scar. Single transfection of FAK Y407F or Src Y529E ameliorated ECM deposition only to some extent (~25%) (Fig. 6F), while co-transfection with these two mutants remarkably reduced ECM deposition by ~60% (Fig. 6F). Masson staining has been commonly used for detecting collagens in various tissues. In this study we performed Masson staining to observe collagen expression and its arrangement in scars after transfection of various constructs. Results revealed that scar tissue transfected with empty vector exhibited massive and disordered collagen fibers (Fig. 6G). Single transfection of FAK Y407F or Src Y529E ameliorated collagen arrangement only to some extent (Fig. 6G), while co-transfection of these two mutants remarkably improved the texture of scar tissue, exhibiting significantly lightened collagen staining and more organized arrangement (Fig. 6G).

### Inhibiting the formation of FAK-Src complex by glabridin reduces ECM protein expression and improves collagen fiber arrangement

**Figure 7:**
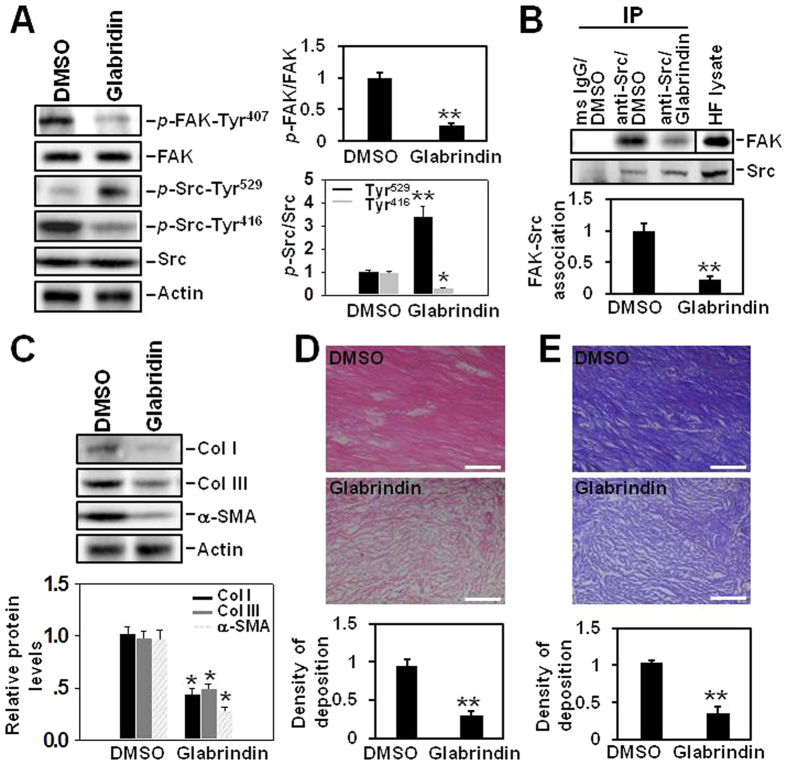
The effect of glabridin on the expression, deposition and arrangement of extracellular matrix in hypertrophic scar. (**A**) Immunoblotting analysis on the expression of *p*-FAK-Tyr^407^, FAK, *p*-Src-Tyr^529^, *p*-Src-Tyr^416^ and Src after treating hypertrophic scar fibroblasts with 100 μM glabridin. Each data point of the phosphorylated form was normalized against its corresponding total protein level with the value in DMSO (vehicle control) group arbitrarily set at 1. Each bar represents a mean ± SD of *n* = 4. ^*^P < 0.05, ^**^P < 0.01. (**B**) Co-immunoprecipitation was performed to examine the effect of glabridin on FAK-Src association in hypertrophic scar fibroblasts. Mouse origin anti-Src IgG was used as the precipitating antibody to extract interacting proteins binding to Src. Normal mouse (ms) IgG was used to replace anti-Src IgG and served as negative control, while HF lysates without co-immunoprecipitation served as positive control. Each data point of FAK was normalized against its corresponding Src level with the value in DMSO group arbitrarily set at 1. Each bar represents a mean ± SD of *n* = 4. ^**^P < 0.01. (**C**) Immunoblotting analysis on the expression of Col I, Col III and α-SMA after treating hypertrophic scar fibroblasts with 100 μM glabridin. Each data point was normalized against its corresponding actin level with the value in DMSO group arbitrarily set at 1. Each bar represents a mean ± SD of *n* = 4. ^*^P < 0.05. (**D**) H&E staining showed ECM deposition in hypertrophic scar tissue after glabridin treatment. Scale bar = 100 μm. The relative density of ECM deposition was analyzed by Image J software with the value in DMSO group arbitrarily set as 1. Each bar represents a mean ± SD of *n* = 4. ^**^P < 0.01. (**G**) Masson staining showed collagen deposition as well as its arrangement. Scale bar = 100 μm. The relative density of collagen deposition was analyzed by Image J software with the value in DMSO group arbitrarily set as 1. Each bar represents a mean ± SD of *n* = 4. ^**^P < 0.01.

Glabridin, a chemical that was found able to block the formation of FAK-Src complex and reduced the aggressiveness of cancer cells^17^, was employed in the study to further verify if it would also affect the pathogenesis of hypertrophic scar. Immunoblotting results showed that glabridin deactivated both FAK and Src in hypertrophic scar fibroblasts as demonstrated by the down-regulated *p*-FAK-Tyr^407^ and *p*-Src-Tyr^416^ protein levels, also the up-regulated *p*-Src-Tyr^529^ level (Fig. 7A). Co-immunoprecipitation revealed glabridin significantly reduced the association between FAK and Src in HS fibroblasts (Fig. 7B). In addition, glabridin remarkably decreased the three major ECM protein Col I, Col III and a-SMA levels in HS fibroblasts (Fig. 7C). When glabridin was used to treat HS tissue, it induced a significant reduction in ECM deposition (Fig. 7D) and made collagen fiber more arranged (Fig. 7E). These findings collectively further suggested that disruption of FAK-Src complex probably through the deactivation of FAK and Src would improve HS pathology.

## Discussion

Hypertrophic scar is pathologically a significant skin fibrotic disease and the major characteristics of HS are the over-proliferation and activation of dermal fibroblasts, as well as the metabolic disorder of collagen-based ECM proteins^19^. Statistically, the incidence of HS ranges from 40~70% following surgery, and up to 91% following burn injury^7^. However, there is currently no effective therapeutically relevant targets for HS treatment largely due to the undefined molecular mechanism.

With the development of molecular biology, more and more protein kinases have been discovered and identified as drug targets to cure diseases, such as Src, FAK, and so on. Src has been involved in various cellular signaling events such as cell proliferation, migration, adhesion to ECM, as well as tumorigenesis. The inhibition of c-Src family was found to suppress the proliferation and migration of dermal fibroblasts induced by TGF-β1, restrain the enhanced expression of α-SMA and the activation of FAK^20^. Moreover, the prohibited activation of fibroblasts and macrophages could further repress scar formation. Src integrates its activity into intracellular signaling networks by either phosphorylation at Tyr^416^, dephosphorylation at Tyr^529^, or through its substrates including FAK^21^,^22^.

FAK is another significant non-receptor protein tyrosine kinase which is expressed ubiquitously with essential roles in cell proliferation and migration. FAK has been implicated in HS formation and development. A recent study has shown that FAK was activated after cutaneous injury^23^, and the fibroblast-specific FAK knockout mice have substantially less inflammation and fibrosis than control mice in a model of hypertrophic scar formation^23^. The phosphorylation of FAK at Tyr^407^ or Tyr^397^ is normally required for FAK activation and various FAK-mediated cell behaviors^24^. In this study, we have identified that FAK and Src were significantly activated in HS *vs*. NS both *in vivo* (Fig. 1) and *in vitro* (Fig. 2), indicated by the up-regulated *p*-FAK-Tyr^407^ and *p*-Src-Tyr^529^ protein levels, as well as the down-regulated *p*-Src-Tyr^529^ level, suggesting the involvement of the activation of these two kinases in HS pathogenesis.

Would healing as well as subsequent scar formation is a process that recruits multiple cell types and various cytokines, such as TGF-β. Our results showed that FAK and Src were significantly activated after TGF-β1 stimulation in normal skin-derived fibroblasts (Fig. 3), further confirming that FAK and Src are involved in TGF-β-mediated signaling pathway in HS pathogenesis.

FAK and Src have been found to trigger the downstream signaling by forming a functional protein complex that is involved in various cellular events, including tumor growth and metastasis, as well as the control of cell shape and focal contact turnover during cell motility. The formation of FAK-Src complex commences with the activation of Src, the activated Src then trans-phosphorylated the kinase domain of FAK, which in turn bands to Src and forms the FAK-Src signaling complex. Our results showed that the interaction between FAK and Src assessed by co-immunoprecipitation (Fig. 4A,B) and their association visualized by dual-labelled immunofluorescence (Fig. 4C) were remarkably enhanced in HS compared to those in NS.

Since HS is a pathologically fibro-proliferative disorder of dermal wound healing characterized by excessive ECM accumulation, the exaggerated deposition and attenuated degradation of type I and type III collagens are the major cause during HS formation. Thus, based on above findings, we were eager to reveal if the abundant collagen deposition could be effectively improved by manipulating the phosphorylated status of FAK and/or Src? Combining site-directed mutagenesis and gene overexpression techniques with FAK Tyr^407^ replaced by non-phosphorylatable phenylalanine (F) and Src Tyr^529^ replaced by phosphomimetic mutant glutamate (E), our results revealed that only simultaneous suppression of FAK activation by overexpressing FAK Y407F mutant and Src activation by overexpressing Src Y529E mutant could obviously down-regulate the expression levels of both Col I (Fig. 5A,F) and Col III (Fig. 5A,G), reduced ECM accumulation (Fig. 6F), and ameliorated collagen arrangement (Fig. 6G), suggesting the synergistic action of FAK and Src on HS pathogenesis.

Src inhibition was found to suppress the accumulation of myofibroblasts and macrophages in the healing wound, and reduce the formation of hypertrophic scar after wound closing in a rabbit ear scar model, without delaying the wound closing process^20^. Our results showed that although the single deactivation of Src by overexpressing phosphomimetic mutant Src Y529E did not affect the protein expression of Col I (Fig. 5A,F) and α-SMA (Fig. 5A,H) *vs.* control (empty vector), it significantly reduced Col III level (Fig. 5A,G), and slightly alleviated ECM deposition (Fig. 6F) and improved the collagen fiber arrangement (Fig. 6G).

The inhibition of FAK was found to effectively reduce α-SMA expression in human fibroblasts and scar formation *in vivo*^23^. FAK was also deactivated in xiamenmycin-induced inhibition of profibrotic effects^25^. A recent study showed that FAK RNAi in HS fibroblasts decreased the expression of TGF-β and α-SMA, inhibited cell proliferation, and reduced collagen synthesis^26^. Our results showed that the single deactivation of FAK by overexpressing the non-phosphorylatable mutant FAK Y407F slightly but significantly down-regulated Col III expression *vs.* control (empty vector) (Fig. 5A,G), mildly improved collagen fiber arrangement (Fig. 6G) and alleviated ECM deposition (Fig. 6F). However, this did not affect α-SMA level (Fig. 5A,H), even more, it induced a surge of Col I level (Fig. 5A,F).

In keloid, another kind of skin scar, the distribution of focal adhesion complex observed by FAK immunofluorescence staining was different from that in control fibroblasts^27^, suggesting FAK might play a general role in skin fibrotic diseases. Moreover, in addition to well-established roles of Src in tumorigenesis, Src performs a proinflammatory function by stimulating intracellular signal transduction, leading to acute inflammatory responses, which is one of the key steps in would healing process. It is likely that the interplay between FAK-Tyr^407^ and Src-Tyr^529^ in regulating HS fibrosis may involve other yet to be identified regulatory partners, such as c-Yes, MAPKs. Further investigation is warranted to identify additional phosphorylation events and the signaling inputs from downstream molecules that are involved. In summary, this report demonstrates that the involvement of FAK/Src activation in HS pathogenesis, which further results in a de-association of FAK-Src complex, produces synergistic effects on regulating ECM deposition and collagen arrangement. These findings thus suggest FAK-Src complex as a potential molecular target, and disrupting FAK-Src association by manipulating FAK/Src activation status might be an effective method to treat HS or related fibrotic diseases.

## Materials and Methods

### Ethics statement

The use of human sample (including scar and normal skin tissues) was conducted under the protocol (No: XJYYLL-2013190) reviewed and approved by Institutional Ethical Committee of the Fourth Military Medical University (FMMU), and the experiments were carried out in accordance with the approved guidelines.

### Antibodies

All antibodies were obtained commercially and the appropriate working dilutions are listed in Table 1.

### Cell culture and treatment

Hypertrophic scar and surrounding normal skin tissues were obtained from six patients (age ranges from 18- to 45-year old) undergoing plastic surgery between Jun 2013 to Jun 2014 in Department of Burns and Cutaneous Surgery in Xijing Hospital (Xi’an, China). Before the experiment, all patients were informed about the purpose and procedures of the study and voluntarily agreed to provide both scar and normal skin tissues. Written consent was obtained from all participants, and all protocols were approved by the Ethics Committee of Xijing Hospital, which is affiliated with the FMMU. Patients received no treatment before skin excision. The dermal portion from scar and normal skin was minced and incubated in a solution of collagenase type I (Sigma–Aldrich, St. Louise, MO) at 0.1 mg/ml at 37 °C for 3 h to isolate fibroblasts. Cells were then pelleted and grown in Dulbecco’s modified Eagle’s medium (DMEM) (Gibco, Grand Island, NY) supplemented with 10% fetal calf serum (Gibco), 100 U/ml penicillin and 100 U/ml streptomycin, and cultured at 37 °C in a 5% (v/v) CO_2_ humidified atmosphere. Normal skin-derived fibroblasts with 70~80% confluence were incubated for 12 h in serum-depleted medium and then treated with TGF-β1 at 5 ng/ml (PeproTech, Rocky Hill, NJ). 24 h thereafter, total proteins from fibroblasts in each sample were prepared for western blot analysis. Glabridin was purchased from Dalian Meilun Biotechnology Co. Ltd. (Dalian, China. Cat# 59870-68-7, Lot# N0706AS), dissolved in DMSO and used at 100 μM as final working concentration to treat either HS fibroblasts or tissues.

### Western blot analysis

Tissues or fibroblasts were washed with ice-cold phosphate-buffered saline (PBS) and lysed using RIPA buffer supplemented with protease and phosphatase inhibitor mixtures (Heart Biological Technology Co. Ltd., Xi’an, China) on ice. Lysates were separated by centrifugation at 4 °C and 14000 × *g* for 10 min. Protein concentration was determined by BCA assay (Pierce, Rockford, IL). 50 μg total protein in each sample was subjected to sodium dodecyl sulfate-polyacrylamide gel electrophoresis (SDS-PAGE) and transferred onto PVDF membranes (Millipore, Bedford, MA). After blocking with 5% non-fat milk, membranes were incubated with specific antibody at appropriate dilutions as listed in Table 1. On the next day, membranes were incubated with horseradish peroxidase (HRP)-conjugated secondary antibody at 1:2000 dilution at 37 °C for 1 h. Then the immunoreactive proteins were visualized by using ECL western blotting detection reagents (Millipore, Billerica, MA) and detected by using MultiImage Light Cabinet Filter Positions (Alpha Innotech, San Leandro, CA).

### Co-immunoprecipitation (Co-IP)

Lysates from normal skin and hypertrophic scar tissues or their corresponding fibroblasts were prepared in immunoprecipitation (IP) lysis buffer (10 mM Tris, 0.15 M NaCl, 1% NP-40, and 10% glycerol, pH 7.4 at 22 °C) supplemented with protease and phosphatase inhibitor cocktails (Sigma–Aldrich) according to the manufacturer’s instructions as described earlier^28^. 500 μg tissue lysates or 300 μg fibroblast lysates for each sample were first pre-cleaned with 2 μg normal rabbit IgG and incubated for 1 h before being precipitated with 10 ml protein A/G agarose beads (Santa Cruz Biotechnology, Santa Cruz, CA) for 1 h, then the supernatant was collected by centrifuging at 1000 × *g* for 5 min. This pre-cleaning step removed non-specific interacting proteins from lysates. Thereafter, the obtained lysates were incubated with 2 μg normal rabbit IgG as negative control or specific rabbit anti-Src antibody for Co-IP on a Labnet Mini LabRoller Rotator (Labnet, Edison, NJ) at 4 °C overnight, to be followed by the incubation with 20 ml protein A/G agarose beads to extract the immunocomplexes. Thereafter, beads were washed with IP lysis buffer three times and the immunocomplexes were extracted in SDS sample buffer at 100 °C followed by immunoblot analysis.

### Immunohistochemistry

Normal skin and hypertrophic scar tissues were fixed in 10% neutral buffered formalin solution overnight, then embedded in paraffin and cut at 4-μm thickness. Sections were quenched with 3% hydrogen peroxide, blocked with 1% bovine serum albumin (BSA), then incubated with specific primary antibody at appropriate dilution (as indicated in Table 1) overnight, followed by incubation with HRP-conjugated secondary antibody for 1 h. DAB was used as the chromogenic agent, and hematoxylin was used for nuclei counterstaining.

### Immunofluorescence staining

Normal skin and hypertrophic scar tissue sections or fibroblasts were fixed with 4% paraformaldehyde for 10 min, permeabilized with Triton X-100 for 10 min, and blocked with 1% BSA for 1 h at room temperature. Cells were then incubated with specific anti-target protein antibody at 4 °C overnight, followed by incubation with Alexa Fluor^®^ 488- (*green*) and/or 555- (*red*) conjugated secondary antibody (Invitrogen, Carlsbad, CA) for 1 h. DAPI was used for nuclear staining. Images were digitally captured by the FSX100 Bio Imaging Navigator (Olympus, Tokyo, Japan) and analyzed by Image-Pro Plus 6.0 system (Media Cybernetics, Silver Spring, MD).

### Construction of mutant FAK and Src mammalian expression vectors

The full-length coding sequences of wild-type human FAK (GenBank accession no. L13616.1) and Src (GenBank accession no. NM_005417.4), including start and stop codons, were amplified from human hypertrophic scar-derived fibroblast cDNA by PCR using primers listed in Table 2, and they were individually cloned into the *Kpn*I/*Xho*I sites of the pcDNA3.1(+) Mammalian Expression Vector (Invitrogen). The mutation of FAK-Tyr^407^ or Src-Tyr^529^ to phenylalanine (Phe) or glutamic acid (Glu), respectively, was performed by three-step mutagenic PCR^29^, using the wild-type FAK or Src construct as the template. Each desired mutation was introduced by the amplification of two overlapping portions of FAK or Src using two primer pairs (a mutagenic primer paired with a flanking primer with the opposite orientation; Table 2). The two PCR products were then fused together by overlap extension^29^, from which the full-length cDNA was amplified with primers listed in Table 2. The cDNA encoding the full-length FAK, Src, or their corresponding mutant FAK Y407F or Src Y529E was individually cloned into pcDNA3.1(+) Mammalian Expression Vector. All sequences were verified by direct DNA sequencing to confirm the full-length and mutant cDNA constructs. For the transfection into hypertrophic scar-derived fibroblasts, plasmid DNA was prepared with a HiSpeed Plasmid Midi Kit (Qiagen, Hilden, Germany).

### Overexpression of wild-type and mutant FAK and Src in hypertrophic scar fibroblast cultures

Hypertrophic scar fibroblasts were isolated from the dermal portion of scar tissues and plated in 60-cm^2^ dishes. Fibroblasts that cultured between 3^rd^ to 4^th^ generation were used in this study. After 24 h-incubation, cells were transfected with: 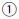 empty vector group: 5 μg pcDNA3.1(+); 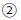 pcDNA3.1(+)/FAK group: 2.5 μg pcDNA3.1(+)/FAK + 2.5 μg pcDNA3.1(+); 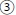 pcDNA3.1(+)/FAK Y407F group: 2.5 μg pcDNA3.1(+)/FAK Y407F + 2.5 μg pcDNA3.1(+); 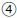 pcDNA3.1(+)/Src group: 2.5 μg pcDNA3.1(+)/Src + 2.5 μg pcDNA3.1(+); 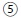 pcDNA3.1(+)/Src Y529E group: 2.5 μg pcDNA3.1(+)/Src Y529E + 2.5 μg pcDNA3.1(+); or 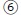 [pcDNA3.1(+)/FAK Y407F + pcDNA3.1(+)/Src Y529E] group: 2.5 μg pcDNA3.1(+)/FAK Y407F + 2.5 μg pcDNA3.1(+)/Src Y529E, by using Lipofectamine 2000 Transfection Reagent (Invitrogen). Western blot analysis was carried out after an additional 72 h.

### *Ex vivo* tissue culture of hypertrophic scar

The *ex vivo* tissue culture of hypertrophic scar was performed as described previously^30^. Briefly, the scar tissue was cut into 10 × 10 mm block, and then transfected with: 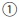 empty vector group: 20 μg pcDNA3.1(+); 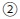 pcDNA3.1(+)/FAK Y407F group: 10 μg pcDNA3.1(+)/FAK Y407F + 10 μg pcDNA3.1(+); 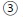 pcDNA3.1(+)/Src Y529E group: 10 μg pcDNA3.1(+)/Src Y529E + 10 μg pcDNA3.1(+); or 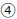 [pcDNA3.1(+)/FAK Y407F + pcDNA3.1(+)/Src Y529E] group: 10 μg pcDNA3.1(+)/FAK Y407F + 10 μg pcDNA3.1(+)/Src Y529E, by using Lipofectamine 2000 transfection reagent (Invitrogen) in a 100 μl volume. This injection was performed intra-dermally into scar tissues. Scar tissues were incubated for 7 days with the culture medium exchanged daily. Tissues were then fixed in 4% paraformaldehyde, followed by routine H&E or Masson trichrome staining according to standard protocols as described earlier^31^. The quantification of ECM and collagen fiber deposition has been analyzed by Image J software.

### Statistical analysis

Statistical analysis was performed using SPSS program, version 13.0. In cases *n* = 4, Mann-Whitney U test was used for paired comparison (Figs 1, 2, 3, 4 and 7), Kruskal-Wallis was used for the comparison among four groups (only selected comparisons were shown) (Fig. 6F,G). In cases *n* = 5, after data were tested and satisfied for normal distribution and equal variance, one-way ANOVA was used and followed by Newman–Keuls test for comparisons among six (Fig. 5) or four groups (Fig. 6A–E) (only selected comparisons were shown). *P* < 0.05 was considered statistically significant.

## Additional Information

**How to cite this article**: Su, L. *et al*. Simultaneous deactivation of FAK and Src improves the pathology of hypertrophic scar. *Sci. Rep.* **6**, 26023; doi: 10.1038/srep26023 (2016).

## Figures and Tables

**Table 1:** Summary of primary antibodies used in this study.

**Target protein**	**Cat no.**	**Lot no.**	**Host**	**Vendor**	**Dilution**			
				**IB**	**IHC**	**IF**	**IP**	
*p*-FAK-Tyr^407^	44650 G	0400#8 A	Rabbit	Invitrogen	1:1000	1:50	1:100	
FAK	sc-558	E1513	Rabbit	Santa cruz biotechnology	1:200	1:50	1:100	
*p*-Src-Tyr^529^	ab32078	YH080908D	Rabbit	Abcam	1:1000	1:50	1:100	
*p*-Src-Tyr^416^	6943 s	1	Rabbit	Cell signaling technology	1:1000			
Src	sc-8056	F3011	Mouse	Santa cruz biotechnology	1:200	1:50	1:100	1:50
Col I	ab96723	GR190879-1	Rabbit	Abcam	1:1000			
Col III	ab7778	NBP1-05119	Rabbit	Abcam	1:1000			
α-SMA	ab7817	1184-1	Rabbit	Abcam	1:1000			
Actin	sc-1616	B2013	Goat	Santa cruz biotechnology	1:500			

IB, immunoblotting; IHC, immunohistochemistry; IF, immunofluorescence; IP, immunoprecipitation.

**Table 2:** Primers used for the construction of different FAK and Src expression vectors.

**Target sequence/mutation**	**Accession no.**	**Primer orientation**	**Primer sequence (5′ → 3′)**	**Nucleotide position**
Primers for the amplification of full-length human FAK and Src				
FAK	L13616.1	S	ACGGTACCATGGCAGCTGCTTACCTTGA	233–252
	L13616.1	AS	ACCTCGAGTCAGTGTGGTCTCGTCTGCC	3372–3391
Src	NM_005417.4	S	ACGGTACCATGGGTAGCAACAAGAGCAA	450–469
	NM_005417.4	AS	ACCTCGAGCTAGAGGTTCACCCCGGGCT	2041–2060
Mutagenic primers used during three-step mutagenic PCR of FAK or Src				
FAK Tyr^407^ → Phe	L13616.1	S	TATAGATGAAGAAGATACTTTTACCATGCCCTCAACCAGGG	1432–1472
	L13616.1	AS	CCCTGGTTGAGGGCATGGTAAAAGTATCTTCTTCATCTATA	1432–1472
Src Tyr^529^ → Glu	NM_005417.4	S	CACGTCCACCGAGCCCCAGGAACAGCCCGGGGAGAACCTCT	2018–2058
	NM_005417.4	AS	AGAGGTTCTCCCCGGGCTGTTCCTGGGGCTCGGTGGACGTG	2018–2058
Flanking primers used during three-step mutagenic PCR of FAK or Src				
pcDNA3.1(+)	EF550208.1	S	TAATACGACTCACTATAGGGAGA	863–885
FAK	L13616.1	AS	ACCTCGAGTCAGTGTGGTCTCGTCTGCC	3372–3391
Src	NM_005417.4	AS	ACCTCGAGCTAGAGGTTCACCCCGGGCT	2041–2060

Sequences marked by dotted underlining represent the restriction enzyme recognition sites (GGTACC for Kpn I; CTCGAG for Xho I) added for cloning into pcDNA3.1(+) Mammalian Expression Vector. Sequences marked by solid underlining represent sites of mutation on FAK or Src. S, sense; AS, antisense. ATG, start codon; TCA or CTA, stop codons.
